# Long-COVID autonomic syndrome in working age and work ability impairment

**DOI:** 10.1038/s41598-024-61455-y

**Published:** 2024-05-23

**Authors:** Luca Rinaldi, Stefano Rigo, Margherita Pani, Andrea Bisoglio, Kareem Khalaf, Maura Minonzio, Dana Shiffer, Maria Angela Romeo, Paolo Verzeletti, Michele Ciccarelli, Maria Grazia Bordoni, Saverio Stranges, Elio Riboli, Raffaello Furlan, Franca Barbic

**Affiliations:** 1 Occupational Medicine, IRCCS Salvatore Maugeri, University of Pavia, Pavia, Italy; 2 Department of Biomedical Sciences, Humanitas University, Pieve Emanuele, Milan, Italy; 3 Department of Neurosurgery, University Vita e Salute S. Raffaele, Milan, Italy; 4 Department of Gastroenterology, St. Michael’s Hospital, University of Toronto, Toronto, Canada; 5 IRCCS Humanitas Research Hospital, Internal Medicine, 20089 Rozzano, Italy; 6 Cardio Calm Srl, Montichiari, Brescia, Italy; 7 Departments of Epidemiology and Biostatistics, Family Medicine and Medicine, Western University, London, ON Canada; 8 Department of Clinical Medicine and Surgery, University of Naples Federico II, Naples, Italy; 9 School of Public Health, Imperial College London, London, UK

**Keywords:** Cardiology, Health occupations, Medical research, Signs and symptoms

## Abstract

Long-COVID19 has been recently associated with long-sick leave and unemployment. The autonomic nervous system functioning may be also affected by SARS-CoV-2, leading to a chronic autonomic syndrome. This latter remains widely unrecognized in clinical practice. In the present study, we assessed the occurrence of Long-COVID19 Autonomic Syndrome in a group of active workers as well as the relationships between their autonomic dysfunction and work ability. This prospective observational study was conducted during the 2nd wave of the pandemic in Italy. Forty-five patients (53.6 ± 8.4 years; 32 M) hospitalized for COVID19, were consecutively enrolled at the time of their hospital discharge (T0) and followed-up for 6 months. Autonomic symptoms and work ability were assessed by COMPASS31 and Work Ability Index questionnaires at T0, one (T1), three and six (T6) months after hospital discharge and compared to those retrospectively collected for a period preceding SARS-CoV-2 infection. Clinical examination and standing test were also performed at T1 and T6. One in three working-age people developed a new autonomic syndrome that was still evident 6 months after the acute infection resolution. This was associated with a significant reduction in the work ability. Recognition of Long-COVID19 Autonomic Syndrome may promote early intervention to facilitate return to work and prevent unemployment.

## Introduction

“Long-COVID19” is a syndrome characterized by symptoms and signs that a patient can experience four or more weeks after contracting SARS-CoV-2 infection, either in a clinically symptomatic form or in a asymptomatic or pauci-symptomatic form^1,2^.

This syndrome includes a wide range of symptoms and signs including fatigue (the most commonly reported, and which is exacerbated by physical effort)^3,4^, dyspnea^5^, tachycardia and dizziness while standing^6^, myalgia and joint pain^7^, difficulty in concentrating^8^, chest pain^8^, diarrhea^9^, sleep disturbances^10^, mood changes^10,11^, and anxiety^10^. Patients may report an overall reduction in perceived quality of life^8,12,13^. Long COVID may affect the workforce and cause loss of job and unemployment^14^, as recently reported. Indeed, in a large population-based study involving more than 15,000 patients with prior COVID-19 infection in US, Long COVID significantly reduced the return to work and reduced the likelihood of working full-time in adjusted analysis^14^. Cognitive effects and fatigue were the main reasons reported by the patients that were unable to return to their previous jobs^15,16^.

Interestingly, several symptoms characterizing Long-COVID19 mirror the Postural Orthostatic Tachycardia Syndrome [POTS]^6,17–19^ that represents the most common form of dysautonomia causing orthostatic intolerance and afflicting nearly 3 million Americans^17^. This observation suggests that SARS-CoV-2 may affect Autonomic Nervous System [ANS] functioning and persist for several months^20,21^, leading to chronic autonomic syndrome. The onset and persistence of autonomic symptoms after the resolution of an acute infection were previously described in literature^22–24^. Of interest, previous studies reported that autonomic dysfunction may negatively impact on work ability^25,26^.

The autonomic symptoms reported after SARS-CoV-2 infection are often underestimated by patients and unrecognized by treating physicians. For this reason, no data is available on the occurrence of chronic autonomic disorders consequent to SARS-CoV-2 infection, particularly in the working-age population.

We performed a prospective study among active workers who were hospitalized with COVID19 during the second wave of pandemic in Italy and followed them up for 6 months after their hospital discharge. An ad-hoc clinical protocol was used to assess autonomic dysfunction symptoms in these patients, to quantify the occurrence of a potential Long-COVID19 autonomic syndrome and the impact on their work ability.

## Results

### Demographic and clinical features

In Table 1 demographic and clinical features of study participants are displayed for the overall sample, subgroups A and B at the time of enrollment (T0). The mean age of study participants was 53.6 ± 8.4 years, they were mostly men, and the mean body mass index was in the overweight range (28.3 ± 4.0 kg/m^2^). Some comorbidities were present among these patients before SARS-CoV-2 infection. The most common were hypertension and diabetes mellitus. The most used medicaments at enrollment were proton pump inhibitors, antihypertensive drugs, beta blockers, statins, hypoglycemic drugs, anticoagulants, and anti-inflammatory drugs. Of note, 13% of the cohort was admitted to the Intensive Care Unit during their hospital stay. The prevailing job-task demand was mental or mixed mental/physical. No significant differences were observed on demographics and clinical features between subgroups A and B.

**Table 1:** Demographic and clinical features of entire cohort, subgroups A and B.

Demographic and clinical features	Entire cohort (N = 45)	Subgroup A (N = 15)	Subgroup B (N = 30)
Age (years)	53.6 ± 8.4	52.8 ± 9.4	54.1 ± 8.1
Sex M/F	32/13	10/5	22/8
BMI (kg/m^2^)	28.3 ± 4.0	28.3 ± 3.95	28.4 ± 4.1
Comorbidities (%)			
Hypertension	6 (13)	3 (20)	3 (10)
Structural heart disease	0 (0)	0 (0)	0 (0)
Arrythmias	1 (2)	1 (7)	0 (0)
Cerebrovascular disease	1 (2)	1 (7)	0 (0)
Neurological disease	1 (2)	0 (0)	1 (3)
Diabetes mellitus	6 (13)	1 (7)	5 (17)
COPD	0 (0)	0 (0)	0 (0)
Neoplasm	3 (7)	0 (0)	3 (10)
Other	15 (33)	2 (13)	4 (13)
Medical therapy (%)			
Beta blockers	8 (18)	3 (20)	5 (17)
ACE-I or ARB	5 (11)	1 (7)	4 (13)
Statins	6 (13)	2 (13)	4 (13)
Anticoagulation	5 (11)	1 (7)	4 (13)
Anti-platelet	2 (4)	0 (0)	2 (7)
Calcium channel blockers	6 (13)	2 (13)	4 (13)
Diuretic	1 (2)	0 (0)	1 (3)
Diabetes drugs	6 (13)	1 (7)	5 (17)
Proton pump inhibitors	10 (22)	1 (7)	9 (30)
NSAIDs	5 (11)	2 (13)	3 (10)
Corticosteroids	5 (11)	2 (13)	3 (10)
Anti-arrhythmic	0 (0)	0 (0)	0 (0)
Other	8 (18)	3 (20)	6 (20)
Hospital stays (%)			
Length of stay > 14 days	10 (22)	3 (20)	7 (23)
Length of stay > 20 days	5 (11)	1 (7)	5 (17)
ICU admission	6 (13)	1 (7)	5 (17)
Job task demand (%)			
Prevalent mental	24(53)	9(60)	15(50)
Prevalent physical	7 (15)	3(20)	4(13)
Mental/physical	14(30)	3(20)	11(37)

Values are expressed as mean ± standard deviation for age and BMI, and in absolute values and percentage for the other variables.

*BMI* body mass index, *COPD* chronic obstructive pulmonary disease, *ACE-I* angiotensin-converting-enzyme inhibitors, *ARB* angiotensin-receptor blocker, *NSAID* non-steroidal anti-inflammatory drugs, *ICU* intensive care unit.

### Time-course of autonomic symptoms and Long-COVID19 autonomic syndrome

The autonomic profile of our population was analyzed through COMPASS-31 scores.

Among the initial 45 subjects, 15 patients met the criteria for Long-COVID19 autonomic syndrome at T6. Therefore, the Long-COVID19 autonomic syndrome at 6 months occurred in 33.3% of enrolled patients. The remaining subjects (N = 30) comprised subgroup B according to our criteria and they were considered as an internal control subgroup.

**Figure 1:**
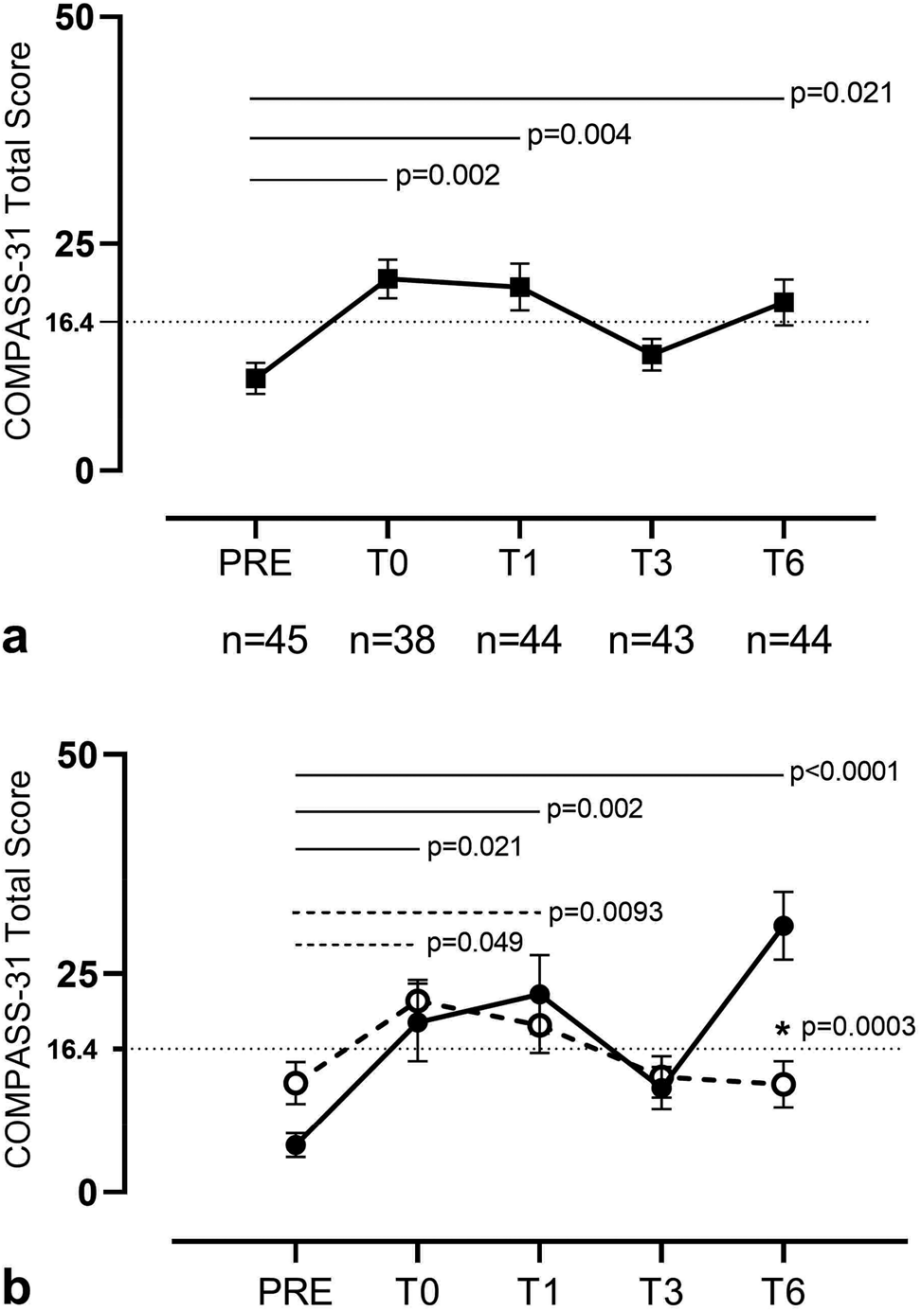
Time course of COMPASS-31 Total Score (0–100) within 6 months from SARS-CoV-2 infection recovery in working-age population. The graphs describe the time course of the total COMPASS-31 scores (mean and standard error) of the entire cohort (**a**), subgroup A (N = 15) and subgroup B (N = 30) (**b**). For the entire cohort the number of patients evaluated at each time point specified in the protocol are reported. Black circle corresponds to subgroup A, while white circle to subgroup B. The dotted line indicates the value of 16.4 COMPASS-31 Total Score corresponding to the cut-off for dysautonomia presence; in panel b, the differences between PRE and time points follow-up are reported for subgroup A (black line) and subgroup B (dashed line); *indicates differences between subgroup A and B at T6. Data are mean ± standard error.

Table 2 and Fig. 1 report the time course of COMPASS-31 total scores for the entire cohort (Fig. 1a) and for A and B subgroups (Fig. 1b) throughout the established time points.

**Table 2:** COMPASS-31 total score values in entire cohort, subgroup A and subgroup B at the follow-up time points.

	PRE	T0	T1	T3	T6
Entire cohort (N = 45)	5.5 (3.1–10.1)	20.4 (10.9–28.6)^#^	15.5 (6.0–32.7)^#^	10.5 (3.6–21.6)	13.9 (5.3–31.4)^#^
Subgroup A (N = 15)	4.8 (3.8–6.0)	16.0 (7.9–27.9)*	21.5 (8.4–30.3)^#^	10.5 (5.4–12.62)*	28.4 (22.2–34.1)^#^
Subgroup B (N = 30)	6.1 (2.6–26.3)	21.2 (14.3–28.6)^#^	12.7 (5.0–32.8)^#^	10.1 (2.6–24.0)	7.3 (2.0–12.7)

Values are expressed as median (interquartile range).

*p < 0.05 follow-up time points vs PRE; ^#^p < 0.01 follow-up time points vs PRE.

A significant increase of total COMPASS-31, indicating a worsening of autonomic functioning, was observed in our cohort from PRE to T6 (p = 0.021) (Table 2; Fig. 1a). The highest intensity of autonomic symptoms was observed at T0 and T1, corresponding to acute and post-acute phase of the disease, respectively. While the intensity of subgroup B’s autonomic symptoms gradually recovered, reaching near-PRE values at T6, subgroup A was characterized by a further increase in symptoms intensity at T1, followed by an initial decline at T3. Thereafter, the COMPASS-31 total score for subgroup A significantly increased at T6 (Fig. 1b). According to the above results, the COMPASS-31 Total Score in subgroup A was significantly higher than subgroup B at T6 (Fig. 1b, Table 3).

**Table 3:** COMPASS-31 total score and single domain scores in subgroup A and B at the follow-up time points.

COMPASS31 domains		PRE	T0	T1	T3	T6
Total score (0–100)	A	4.8 (3.8–6.0)	16.0 (7.9–27.9)	21.5 (8.4–30.3)	10.5 (5.4–12.6)	28.3 (22.2–34.1)
	B	6.1 (2.6–26.3)	21.2 (14.3–28.6)	12.7 (5.0–32.8)	10.1 (2.6–24.0)	7.3 (2.0–12.7)
OI (0–40)	A	0.0 (0.0–0.0)	0.0 (0.0–17.0)	12.0 (0.0–23.0)^#^	0.0 (0.0–4.0)^#^	16.0 (12.0–22.0)^#^
	B	0.0 (0.0–15.0)	12.0 (8.0–20.0)	0.0 (0.0–16.0)	4.0 (0.0–16.0)	0.0^§^ (0.0–8.0)
Vasomotor (0–5)	A	0.0 (0.0–0.0)	0.8 (0.0–1.7) *	0.0 (0.0–1.2)	0.0 (0.0–0.0)	0.0 (0.0–0.0)
	B	0.0 (0.0–0.0)	0.0 (0.00–1.7)	0.0 (0.0–0.0)	0.0 (0.0–0.0)	0.0 (0.0–0.0)
Secretomotor (0–15)	A	2.1 (0.00–2.1)	4.4 (1.6–6.4)	4.3 (2.1–6.4)	4.3 (0.0–5.4)	2.1 (0.0–7.6)
	B	2.1 (0.0–2.1)	4.3 (2.1–4.3)	2.1 (0.0–6.4)	0.0 (0.0–2.1)	0.0 (0.0–4.3)
Gastrointestinal (0–25)	A	1.8 (0.0–3.6)	4.5 (3.3–5.8)	1.8 (0.0–5.8)	0.0 (0.0–2.7)	6.2 (3.1–6.7) *
	B	1.8 (0.0–5.1)	3.1 (1.1–6.0)	2.7 (0.9–6.0)	0.9 (0.0–2.7)	1.8 (0.9–4.5)
Bladder (0–10)	A	0.0 (0.0–0.0)	0.0 (0.0–1.4)	0.0 (0.0–1-1)	0.0 (0.0–1.1)	1.1 (0.0–2.8)^#^
	B	0.0 (0.0–1.1)	0.0 (0.0–0.0)	0.0 (0.0–1-1)	0.0 (0.0–2.2)	0.0 (0.0–2.2)
Pupillomotor (0–5)	A	0.3 (0.0–0.5)	0.5 (0.0–1.6)	0.1 (0.7–1.7)	0.7 (0.0–1.7)	1.3 (0.3–2.7) *
	B	0.3 (0.0–0.9)	0.0 (0.0–1.2)	1.2 (0.1–1.7)	0.0 (0.0–1.7)	0.1 (0.0–1.1)

Values are expressed as median (interquartile range).

*OI* orthostatic intolerance.

*p < 0.05 follow-up time points vs PRE; ^#^p < 0.01 follow-up time points vs PRE; ^§^p < 0.05 B vs A.

Median and interquartile values for COMPASS-31 Total Score and six autonomic function domains in subgroups A and B are reported in Table 3. Of interest, patients reported orthostatic intolerance symptoms during the acute and post-acute phase of the disease (T0 and T1).

In subgroup A, after a mild amelioration reported at T3, an additional marked worsening was reported at T6. The vasomotor domain was altered only during the acute phase of the disease (T0). The gastrointestinal, bladder, and pupillo-motor domains were significantly altered at T6 compared to PRE, thus suggesting a long-term manifestation of these autonomic dysfunctions.

In subgroup B the autonomic symptoms were reported during the acute phase of the disease and then showed a progressive amelioration until T6 (Fig. 1b, Table 3).

### Hemodynamic parameters and autonomic symptoms while supine and during standing at T1 and T6

As reported in Table 4 in the entire cohort, heart rate (HR) increased during active standing compared to supine, both at T1 and T6. While supine, HR was lower at T6 compared to T1. At T1, systolic arterial pressure (SAP) values increased during active standing, while remaining unchanged at T6. Diastolic arterial pressure (DAP) increased during active standing both in T1 and T6. Autonomic symptoms assessed by VOSS were reduced both while supine and during standing at T6 compared to T1 thus indicating a global improvement independently by the gravitational stimulus.

**Table 4:** Hemodynamic parameters and VOSS at T1 and T6 while Supine and during Standing in the entire cohort, subgroup A, and subgroup B.

	T1		T6	
	Supine	Standing	Supine	Standing
Entire cohort	N = 44		N = 44	
HR b/min	65 (57–73)	75 (68–84)^#^	61 (53–70)*	72 (64–82)^#^
SAP mmHg	120 (115–130)	128 (120–137)^#^	122 (115–126)	123 (118–130)
DAP mmHg	74 (66–80)	81(75–90)^#^	72 (70–75)	80 (76–85)^#^
SpO2	97 (96–97)	98 (96–99)	97 (96–98)	98 (97–99)
RR breath/min	15 (13–19)	16 (15–19)	15 (13–17)	16 (15–19)
VOSS (0–90)	0 (0–4)	1 (0–6)	0 (0–0) *	0 (0–1) *
Subgroup A	N = 15		N = 15	
HR b/min	59 (56–72)	71 (67–87)^#^	58 (50–65) *	70 (62–77)^#^*
SAP mmHg	121 (111–130)	122 (117–132)	122 (115–125)	122 (118–129)
DAP mmHg	72 (63–80)	77 (69–86)	70 (70–77)	80 (78–84)^#^
SpO2	97 (95–97)	98 (96–98)	98 (97–98)	99 (98–99)
RR breath/min	16 (13–20)	16 (15–19)	15 (14–18)	16 (15–19)
VOSS (0–90)	2 (0–8)	4 (0–14)	0 (0–0)	0 (0–2)
Subgroup B	N = 29		N = 29	
HR b/min	68 (58–74)	75 (68–82)^#^	63 (57–75)	75 (67–85)^#^
SAP mmHg	120 (116–129)	130 (120–138)^#^	122 (115–126)	123 (118–135)
DAP mmHg	74 (70–80)	84 (77–91)^#^	72 (70–75)	80 (75–85)^#^
SpO2	97 (96–97)	98 (96–99)	97 (96–98)	97 (96–98.5)
RR breath/min	15 (13–16)	15 (14–17)	15 (13–16)	16 (15–18)
VOSS (0–90)	0 (0–3)	0 (0–4)	0 (0–1)	0 (0–1)

Values are expressed as median (interquartile range).

*HR* heart rate, *SAP* systolic arterial pressure, *DAP* diastolic arterial pressure, *SpO2* oxygen saturation, *RR* respiratory rate.

*p < 0.05 T6 vs T1. ^#^p < 0.05 standing vs supine.

In subgroup A, HR increased during standing compared to supine both in T1 and T6. Heart rate decreased both while supine and standing in T6 compared to T1. At T6, DAP increased during standing compared to supine. The hemodynamics and Vanderbilt Orthostatic Symptom Score (VOSS)^27^ in subgroup B paralleled what was observed in the entire cohort.

### Work ability index time-course and relationship between WAI and COMPASS-31

**Figure 2:**
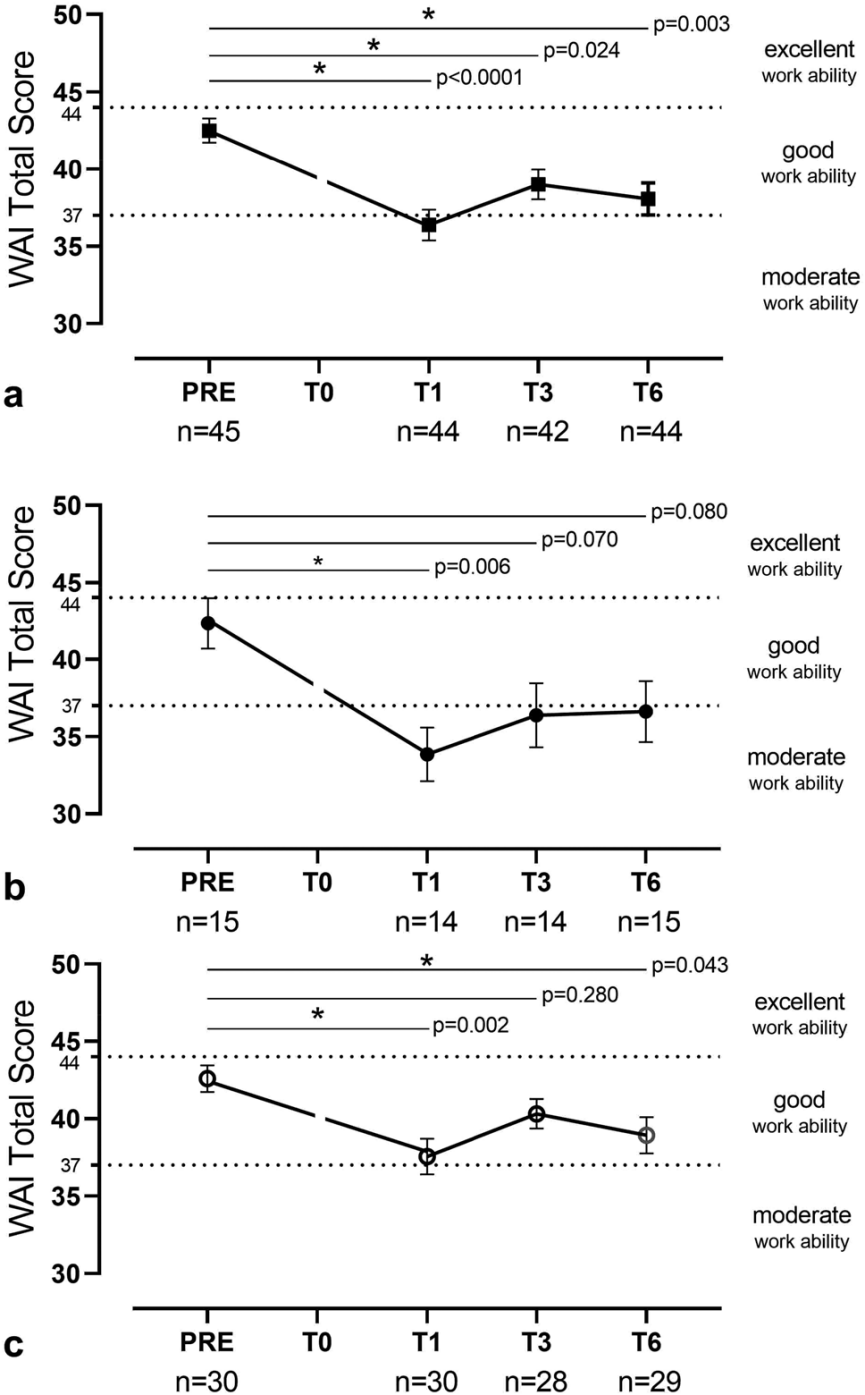
Time course of WAI Total Score (0–49) within 6 months from SARS-CoV-2 infection recovery in working-age population. The graphs show the time-course of WAI total score during the follow-up time points observed in the entire cohort (**a**), in subgroup A (**b**) and in subgroup B (**c**). The WAI total score was markedly reduced at T1 compared to PRE and remained reduced up to T6 in the entire cohort and in two subgroups. Notice that in subgroup A, the mean values of WAI at T6 remains slightly reduced compared to PRE although this difference is not significant. Data are mean ± standard error.

In Fig. 2, the time-course up to 6 months of WAI total scores observed in the entire cohort (a), in subgroup A (b), and in subgroup B (c) are shown. The mean WAI total score was markedly reduced at T1 compared to PRE and remained reduced up to T6 in the entire cohort and in two subgroups. Despite a change of class from “good” to “moderate” work ability in subgroup A at T6 displaying, the WAI values at T6 were only slightly lower than WAI values at PRE.

At T6 the WAI were collected in the 15 patients of subgroup A and in 26 patients of subgroup. The single WAI domains at T6 are reported in eTable 1. Of interest, the subgroup A was characterized by a longer sick leave (3^rd^ interquartile 25–99 days) than the subgroup B (3^rd^ interquartile < 10 days).

**Figure 3:**
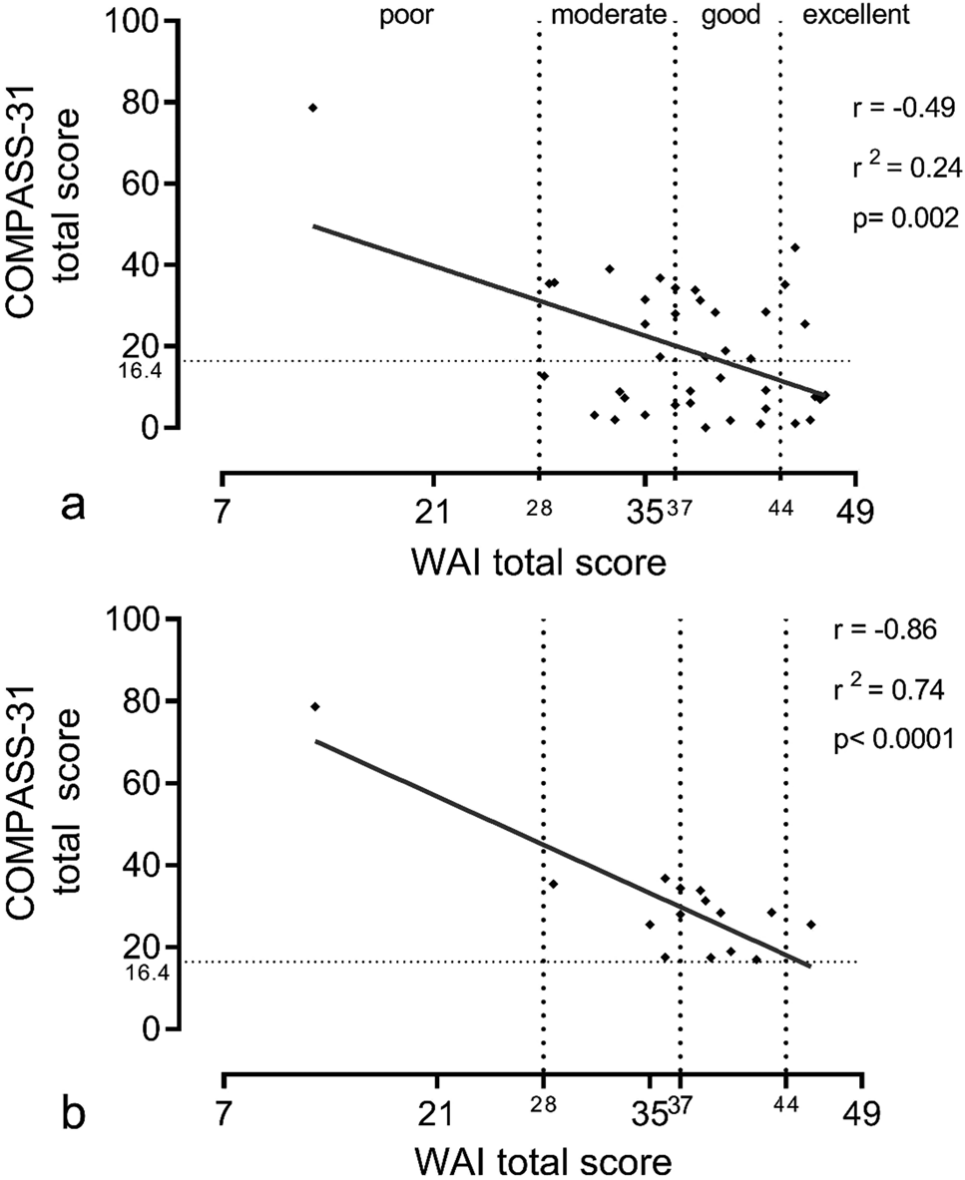
Relationships between COMPASS-31 and WAI total scores. The graphs show the relationship between COMPASS-31 total score and WAI total score in the cohort of 40 patients that completed both COMPASS-31 and WAI questionnaires (**a**) and in subgroup A of patients (**b**). The dotted vertical lines indicate WAI values corresponding to different classification of work ability: poor < 28; moderate 28–37; good 37–44; excellent > 44. Notice that the highest the COMPASS-31 total score the lower the WAI. The inverse relationship between the two scores is more evident in the subgroup A, corresponding only to the patients who developed the Long-Covid autonomic syndrome.

The relationship between the intensity of autonomic symptoms (COMPASS-31 Total Score) and work ability (WAI Total Score) at T6 in all the patients that completed both COMPASS-31 and WAI questionnaires (N = 40) and in patients who developed Long-COVID19 autonomic syndrome is shown in Fig. 3. Of note, the higher the autonomic symptom intensity, the lower the work ability. This inverse relationship between the two scores was stronger in subgroup A (Fig. 3b).

## Discussion

The main results of this prospective observational study indicated that:

1. SARS-CoV-2 infection affected the autonomic nervous system and that a long-lasting autonomic impairment occurred in 33.3% of our working age patients.
2. After 6 months following the acute infection resolution, a compromised work ability was still present in fifteen patients. Their work ability was inversely correlated with the intensity of autonomic dysfunction.

Recent studies have provided evidence that long COVID may be affecting the workforce and that subjects with self-reported Long-COVID symptoms were more likely to be unemployed^14,15^. In this study, we aimed to assess the effects of Long-COVID autonomic syndrome on work ability. Indeed, the integrity of autonomic functions is crucial to guarantee an adequate response to environmental stimuli including physical^25,28^ and mental^29,30^ demands that characterize most of the work environments.

Similarly to what was observed after other acute infections^22–24^, recent studies reported that SARS-CoV-2 may affect the autonomic nervous system^20,21^.

The reduction of Long-COVID symptoms were reported in two patients after stellate ganglion block^31^ thus suggesting a potential role of sympathetic nervous system dysregulation in sustaining Long-COVID syndrome. In addition, the dysregulated neuro-inflammatory state observed in Long-COVID has been attributed to a subtle form of dysautonomia^32^.

In the present study we focused on a selected group of unvaccinated active workers (i.e. people that were fit to work before Sars-Cov2 infection) who developed a severe form of COVID19 during the second pandemic wave in Italy. Some of patients suffered from comorbidities and were quite elderly. The patients were consecutively enrolled at the time of their hospital discharge and followed-up for 6 months to address the occurrence of Long-COVID autonomic syndrome.

This study demonstrated that 1/3 of patients that were in quite good health before COVID19, were affected by Long-COVID autonomic syndrome. Previous studies addressed the prevalence of autonomic abnormality symptoms after COVID19. Larsen et al.^33^ reported that 67% of the large study population had a COMPASS-31 total score higher than 20. However, data were collected through online questionnaires and comprised subjects that either had a diagnosed COVID19 infection or that only manifested COVID19 symptoms without confirmatory laboratory tests. The patients reporting autonomic symptoms at the time point of this evaluation could have had autonomic alterations even before COVID19. In addition, a potential overestimation of findings that characterizes any online surveys should be considered^34^. Other studies focused only on the prevalence of long-lasting autonomic symptoms in selected patients referred to a tertiary dysautonomia center^35^ or a long-COVID outpatient clinic^36^. Shouman et al.^35^ reported autonomic symptoms in 63% of patients enrolled while Boite and colleagues showed the persistence of autonomic symptoms in 61% of patients investigated for Long-COVID^36^. A recent review by Hira et al.^37^ confirmed the high prevalence of autonomic dysfunction among patients with Long-COVID. However, the mechanisms underlying this chronic autonomic syndrome remain poorly understood^37,38^.

In the present study, we further checked the responses to the questionnaires of all the subjects that displayed a significant difference between total COMPASS-31 scores at T3 and T6 (Subgroup A). These suggested that after an initial autonomic symptom improvement following the acute and sub-acute COVID19, a new worsening of autonomic functions occurred in these susceptible patients.

One patient of subgroup B missed T1 and T6 tests, two patients (one of subgroup A and one of subgroup B) were not reachable for the questionnaires at T3. Finally, 4 patients of subgroup B did not provide WAI at T6.

The most common autonomic symptom reported by subgroup A at T6 was the orthostatic intolerance (Table 3) that included fainting, dizziness, brain fog or difficulties in thinking after standing up^39^. The orthostatic intolerance seemed to be absent before the acute infection (PRE), started during the acute phase of the infection, and were still severe six months after the acute phase resolution. In addition, at this time point, the patients reported gastrointestinal, bladder and pupillo-motor alterations that were absent before COVID19, in accordance with previous observations^40–43^.

Patients of subgroup A reported symptoms of orthostatic intolerance during their daily life but the hemodynamic response to the gravitational stimulus was in the normal range (Table 4). In contrast to POTS, a significant orthostatic intolerance was not reported. We can only hypothesize that a mild temporary increase in blood pressure due to a potential white-coat syndrome, that has been described particularly in young patients^44^, might have lessened the orthostatic intolerance symptoms during the standing test.

The present study identified a group of subjects that was susceptible to develop a new onset of chronic autonomic syndrome after COVID19 (Subgroup A). The recognition of autonomic symptoms’ occurrence, often underestimated by the patients and unrecognized by the physicians^21^ may facilitate the patients’ management after COVID19, prevent disability and worsening of pre-existing diseases.

The possibility to distinguish different phenotypes before COVID19 to predict those patients who may develop a Long COVID19 autonomic syndrome is a fascinating challenge. However, this will require wider clinical trials and ad hoc methods of data analysis.

As reported by recent studies, Long-COVID seems to be associated with a higher likelihood of being unemployed, as well as a lower likelihood of working full-time^14,15^. Presently, it is unclear the extent to which Long-COVID may cause unemployment^15^ as well as the role of autonomic impairment in sustaining this work impairment. The cognitive symptoms^15,16^, depression and fatigue^16^ resulted strongly involved in the reduced work capability after COVID19. This may partially explain the slight decline of WAI observed also in subgroup B that do not develop the Long-COVID autonomic syndrome.

In this study, we evaluated the impact of COVID19 on work ability by using the well-validated WAI questionnaire^25,46,47^. This aspect represents a novelty in the studies on COVID19 patients.

Of interest, in subgroup A, the mean values of WAI at T6 shifted from “good” to “moderate” work ability (Fig. 3b). We did not report results of WAI at T0 because at the time of hospital discharge the patients were completely unable to work. A slight decline of WAI has been observed also in the subgroup B that did not develop a chronic autonomic syndrome after COVID19 recovery. However, the WAI of these subjects was similar to what observed before COVID19 and classified “Good” (Fig. 2). It is noticeable that the mental job task demand was the prevalent reported by both subgroups of patients. According with previous wide survey-based studies^15,16^, cognitive impairment may have partially promoted this change in WAI. The reduced sick leave of patients of subgroup B might have concurred to a higher WAI value at T6 of these patients.

In POTS^25^ and Pure Autonomic Failure^26^, we recently described an inverse relationship between the intensity of autonomic symptoms and cardiovascular autonomic changes induced by orthostatic stimulus and work ability. In line with these observations, this study provides evidence for an inverse relationship between the autonomic symptom’s intensity and work ability in Long-COVID autonomic syndrome. Of interest, in patients who developed Long-COVID autonomic syndrome, this relationship was stronger.

Autonomic dysfunction showed a negative impact on patient’s work ability for an extended period. It is important to remember that the alteration in autonomic functions and in particular, orthostatic intolerance may affect cognitive function^29^ as already described in other autonomic disorders such as POTS^17^. Of interest, brain fog or memory impairment and fatigue were reported by almost 46% of respondents to a widely administered survey, in patients with long-COVID^15^.

These observations need to be considered by clinicians, general practitioners and occupational physicians when managing patients who must go back to work after COVID19.

### Limitations and strengths

We acknowledge the limitations of the present study. First, the results cannot be generalizable to the entire working age population. Indeed, we focused on active workers, some of them suffering from comorbidities, and quite elderly. However, as consecutively enrolled at the time of their hospital discharge, our study population provides novel information on new occurrence of Long-COVID autonomic syndrome in this group of active workers.

Furthermore, the small number of subjects represents an additional limitation of the study, partially counteracted by the internal control group. The observed association between individual autonomic symptoms and work ability at T6 of our forty patients does not provide a definitive causal link; however, our study provides important insights in the management of return to work after COVID19.

The assessment of autonomic symptoms and work ability were based on questionnaires, which may be limited by their subjective interpretation. However, it should be noted that the tools used have been previously validated in large populations across different clinical settings^46–48^. Furthermore, the questionnaires were administered by trained researchers that also provided explanations and support to the patients during the survey and a double-check of the answers was performed when it was needed.

Finally, since the vaccination campaign started after the period of enrollment, all the participants were not yet vaccinated and did not receive any vaccination during the first 6-month follow up. Therefore, the occurrence of autonomic symptoms was not influenced by the vaccine^49^.

## Conclusion

Our findings suggest that SARS-CoV-2 infection may affect the autonomic nervous system by inducing long term changes in autonomic functions in working age patients and may negatively impact on their work ability. Searching for autonomic symptoms’ occurrence may facilitate the patients’ management and prevent worsening of pre-existing diseases. While our results are not generalizable to the entire working age population, they provide new insights on the relationship between chronic autonomic dysfunction after COVID19 and work ability and may help health professionals in the field of occupational health to mitigate the risk of unemployment after COVID.

## Methods

The present prospective study was conducted from December 2020 to May 2021 (eFig. 1) at, Humanitas Research Hospital (HRH) in Milan, Italy. The patient’s follow-ups were performed at the Cardiovascular Autonomic Disorders out-patient Unit of HRH that represents a tertiary referral center for the study of cardiovascular autonomic diseases. The study was approved by HRH Ethical Review Board (Approval #956/20). Written informed consent was obtained from all the subjects participating in the study. All procedures performed in the present study were in accordance with the Helsinki declaration.

### Study population

Forty-five patients hospitalized with COVID19 were consecutively enrolled at the time of their hospital discharge (T0). Inclusion criteria were age between 18 and 67 years, an active working status, the ability to maintain the standing position autonomously and the ability to attend scheduled follow-up visits. We excluded patients that were affected by autonomic diseases, dementia, severe cognitive disturbances, atrial fibrillation, and epilepsy before SARS-CoV-2 infection. All the participants were unvaccinated against COVID at the time of study enrolment and did not receive any vaccines throughout the follow-up period.

### Study protocol

**Figure 4:**
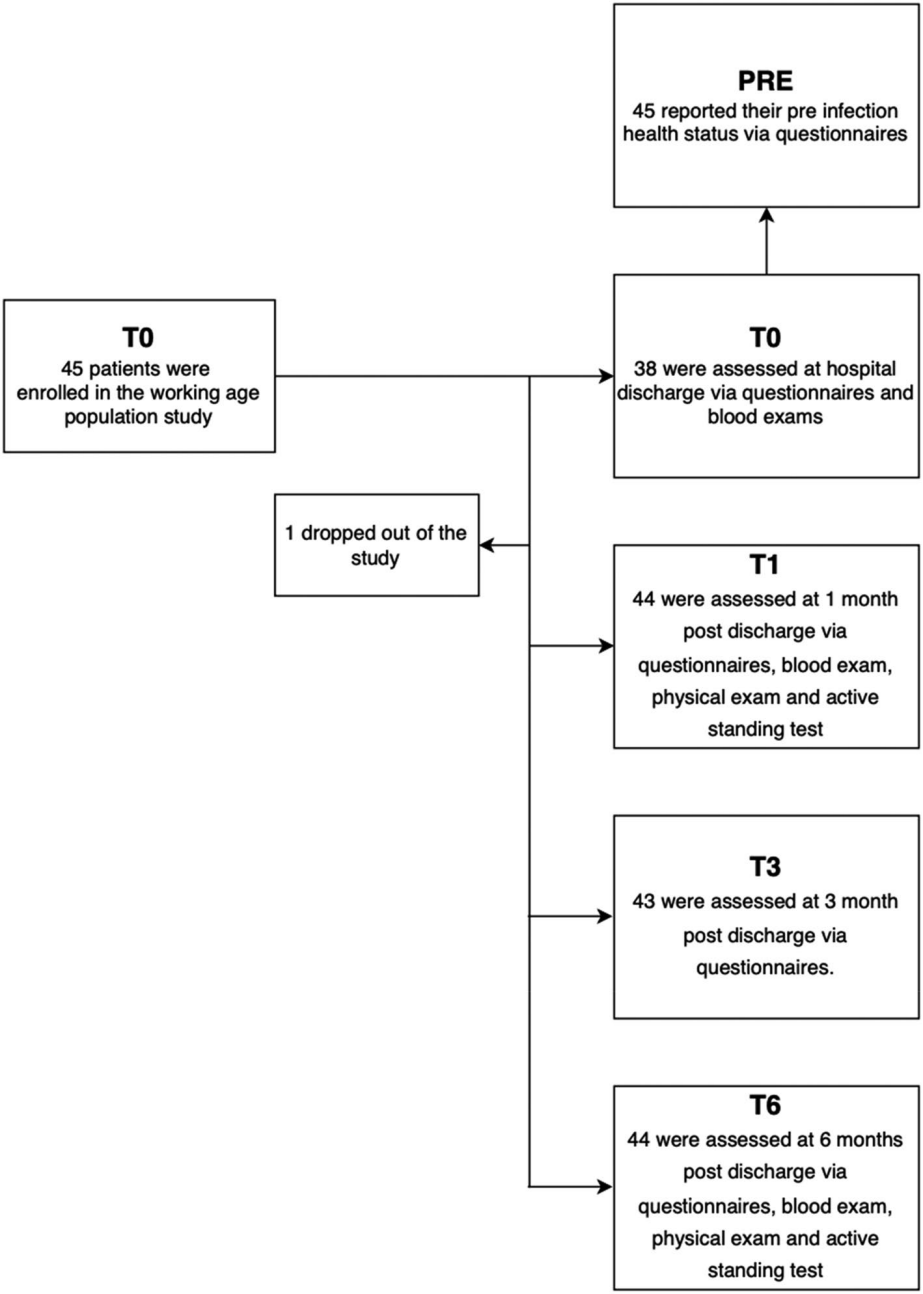
Study protocol. The flow-chart summarizes the study protocol. Only 1 patient dropped out of the study.

At T0, all the patients were evaluated using an anamnestic general data form^50^ and the Composite Autonomic Symptoms Score-31 (COMPASS-31)^39,51^ questionnaire (Fig. 4). The health status prior to SARS-CoV-2 infection and hospitalization (PRE) was retrospectively assessed by the questionnaires described above. Baseline work capability (at PRE) was also evaluated using the Work Ability Index questionnaire^46,47^. One patient missed the T1 and T6 tests, while 2 patients were not reachable for the questionnaires at T3. Finally, 4 patients did not provide WAI at T6.

One month after hospital discharge (T1), the enrolled patients underwent the first clinical follow-up by a physician with expertise in autonomic disorders. The evaluation included questionnaires (COMPASS-31 and WAI), history taking, complete clinical evaluation, and standing test^52^.

After 3 months from hospital discharge (T3), the patients were followed-up telephonically by a trained researcher and all the questionnaires were completed with the information provided by the patients.

At 6 months from hospital discharge (T6), all patients underwent the last follow-up visit of the protocol with the same procedure described above for T1.

### Questionnaires

*COMPASS-31* is a validated questionnaire used to evaluate autonomic symptoms^25,26^. It consists of 31 items which aim to evaluate six domains of autonomic functions (orthostatic intolerance; vasomotor; secretomotor; gastrointestinal; bladder; pupillomotor). The overall score is achieved by calculating the raw domain scores, which are determined by adding up the points of the questions in each domain. The raw score of each domain is multiplied by a weight index which yields the final domain score. The sum of all domain scores produces the total score, which ranges from 0 (normal) to 100 (the most severe symptoms)^39^.

*Work Ability Index* is a validated tool^46,47^, which captures a self-assessment of employees’ work ability. The index considers the demands of work, the worker’s health status and resources. The physician in charge rated the responses according to seven items: 1. current work ability (WA) compared to the lifetime best (range 0–10); 2. WA in relation to the job’s demands (range 2–10); 3. Current diseases diagnosed (range 1–7); 4. Estimated work impairment (range 1–6); 5. Sick leave in the last period due to diseases (range 1–5); 6. Own prognosis of WA 2 years from now (score 1, 4,7); 7. Mental resources (range 1–4). The total WAI score range is 7–49. Ultimately, this tool assigns patients to 4 categories based on their ability to work: poor (7–27), moderate (28–36), good (37–43), and excellent (44–49).

In the present study the patients were carefully instructed to answer all the questionnaires by considering the actual time point of follow-up with respect to PRE.

#### Vanderbilt orthostatic symptom score

The Vanderbilt Orthostatic Symptom Score (VOSS, 0–90)^27^ was used to quantify the potential symptoms of orthostatic intolerance during the standing test. Participants had to score the intensity of symptoms on a scale of 0–10 with 0 corresponding the absence of the symptom, and 10 indicating maximal intensity of the symptom. Symptoms included mental clouding, blurred vision, shortness of breath, rapid heartbeat, tremors, chest discomfort, headache, lightheadedness, and nausea. The VOSS score was obtained by summing the 9 single scores^27^.

### Clinical evaluation and standing test

After a detailed history taking, all the patients underwent a physical examination by the physician in charge and the Standing test^52^. The latter was used to evaluate the hemodynamic and respiratory changes induced by the orthostatic stimulus. The intensity of orthostatic intolerance symptoms was quantified by the Vanderbilt Orthostatic Symptom Score. For each patient, continuous ECG recording by Vivalink^53^, O_2_ saturation by digit pulse-oximeter (NIHON KOHDEN), respiratory frequency and brachial blood pressure values by external automated device were obtained during 10 min supine and 10 min of active standing.

### Subgroups of the study population

To estimate the incidence of Long-COVID autonomic syndrome, we defined two different groups from the 45 enrolled patients.

Previous studies performed on diabetic patients^54^ suggested that a cut-off value of COMPASS-31 total score equal to 16.44 may be used to distinguish between the absence (< 16.44) or presence (> 16.44) of significant autonomic dysfunction. Therefore, we categorized the study population in two different subgroups A and B. Subgroup A included those patients who, on COMPASS-31, scored less than 16.44 at PRE and more than 16.4 at T6, subgroup B included the remaining population i.e., the patients that did not develop any autonomic symptoms following the acute phase of COVID19, including those who reported COMPASS-31 higher than 16.4 at PRE without worsening after COVID19. Therefore, patients of subgroup A had no relevant autonomic dysfunction symptoms before SARS-CoV-2 infection (PRE) and ended up displaying signs of autonomic abnormalities at 6 months from infection resolution (T6). This subgroup represents patients who developed Long-COVID autonomic syndrome as previously defined.

### Statistical analysis

The sample size corresponded to the consecutive working age patients enrolled within the defined enrollment period.

Descriptive data are presented as median and interquartile range for continuous variables, and as numbers and percentages for categorical variables. In Figs. 1 and 2 data are shown as mean ± standard error. The Shapiro–Wilk test was used to determine whether the variables present a Gaussian distribution. For the comparison among the identified subgroups within our cohort at the same time point, the unpaired t-test, Chi-square test, and Fisher exact test were used where appropriate. One-Way ANOVA for repeated measures, followed by Dunnett’s post hoc test, was used to evaluate changes in autonomic symptoms and WAI at different follow-up times compared to PRE. Linear regression analysis was used to evaluate the relationship between COMPASS-31 total score and WAI at T6. A P-value < 0.05 was considered statistically significant. GraphPad PRISM version 9.3.1 was used to perform statistical analysis.

### Supplementary Information

Supplementary Table 1.

Supplementary Figure 1.

## Data Availability

All data generated or analysed during this study are included in this published article [and its supplementary information files].
