# Supplementary material for: Long-COVID autonomic syndrome in working age and work ability impairment
Source: Sci Rep. 2024 May 23;14:11835. doi: 10.1038/s41598-024-61455-y (PMC11116376; doi:10.1038/s41598-024-61455-y)
Supplement: Supplementary file 1 — Supplementary Table 1. [file 41598_2024_61455_MOESM1_ESM.docx]

eTable 1. Work Ability Index Domains at T6 in the subgroups A and B

| **Work Ability Index Domains** | **Subgroup A (N =15)** | **Subgroup B (N = 26)** |
| --- | --- | --- |
| 1. Current work ability compared to the lifetime best (0–10) | 7 (7-8) | 8 (7-9) |
| 1. Work ability in relation to the job demands (2–10) | 8 (8-10) | 9 (8-10) |
| 1. Current diseases diagnosed (1–7) | 5 (5-7) | 5 (4-7) |
| 1. Estimated work impairment (1–6) | 5 (4-6) | 6 (5-6) |
| 1. Sick leave in the last year due to the diseases (1–5) | 2 (1-2) | 3 (2-4) |
| 1. Own prognosis of work ability two years from now (1,4,7) | 7 (7-7) | 7 (7-7) |
| 1. Individual resources (1–4) | 3 (3-4) | 4 (3-4) |

Values are expressed as median (interquartile range). As described in the WAI tool reported in Methods, the highest the score the better the domain. Sick leave duration (5): 1 corresponds > 100 days; 2, 25-99 days; 3, 10-24 days; 4, <10days; 5, no sick leave.
