# Supplementary material for: Long-COVID autonomic syndrome in working age and work ability impairment
Source: Sci Rep. 2024 May 23;14:11835. doi: 10.1038/s41598-024-61455-y (PMC11116376; doi:10.1038/s41598-024-61455-y)
Supplement: Supplementary file 2 — Supplementary Figure 1. [file 41598_2024_61455_MOESM2_ESM.docx]

**Supplementary Figure**

**Time of patient’s enrollment during 2^nd^ wave of pandemic in Italy.**

**
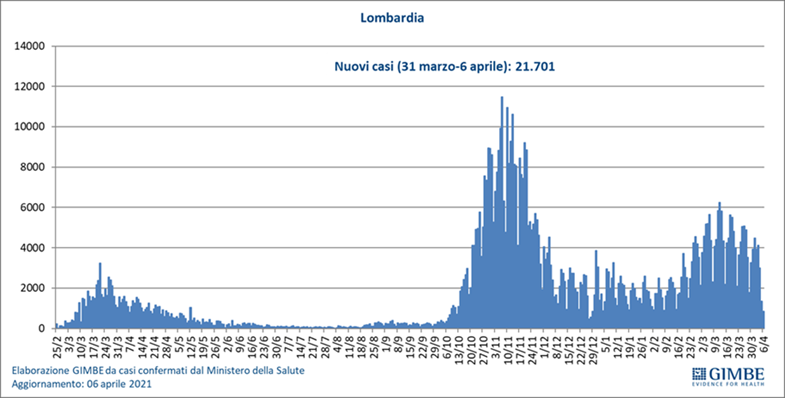
**

Modified by GIMBE (Italian Group Medicine Based Evidence). Sars-Cov2 pandemic waves in Italy from February 2020 to April 2021. The black line indicates the period corresponding to the patient’s enrollment.
